# The FoodCast research image database (FRIDa)

**DOI:** 10.3389/fnhum.2013.00051

**Published:** 2013-03-01

**Authors:** Francesco Foroni, Giulio Pergola, Georgette Argiris, Raffaella I. Rumiati

**Affiliations:** Cognitive Neuroscience Sector, SISSA - TriesteTrieste, Italy

**Keywords:** food, validated images database, food processing, category specificity

## Abstract

In recent years we have witnessed an increasing interest in food processing and eating behaviors. This is probably due to several reasons. The biological relevance of food choices, the complexity of the food-rich environment in which we presently live (making food-intake regulation difficult), and the increasing health care cost due to illness associated with food (food hazards, food contamination, and aberrant food-intake). Despite the importance of the issues and the relevance of this research, comprehensive and validated databases of stimuli are rather limited, outdated, or not available for non-commercial purposes to independent researchers who aim at developing their own research program. The FoodCast Research Image Database (FRIDa) we present here includes 877 images belonging to eight different categories: natural-food (e.g., strawberry), transformed-food (e.g., french fries), rotten-food (e.g., moldy banana), natural-non-food items (e.g., pinecone), artificial food-related objects (e.g., teacup), artificial objects (e.g., guitar), animals (e.g., camel), and scenes (e.g., airport). FRIDa has been validated on a sample of healthy participants (*N* = 73) on standard variables (e.g., valence, familiarity, etc.) as well as on other variables specifically related to food items (e.g., perceived calorie content); it also includes data on the visual features of the stimuli (e.g., brightness, high frequency power, etc.). FRIDa is a well-controlled, flexible, validated, and freely available (http://foodcast.sissa.it/neuroscience/) tool for researchers in a wide range of academic fields and industry.

## The foodcast research image database (FRIDa)

Like other animals, humans need to process information about possible sources of nutrition and to avoid poisoned or uneatable food. The decision to consume a particular food is modulated by factors such as internal cues, nutritional status and immediate energy needs (Ottley, 2000). However, the food-rich environment in which we presently live makes the regulation of food choices a very complex phenomenon that is still poorly understood (Killgore and Yurgelun-Todd, 2006). Understanding how we think about and behave toward food is very important also because of the numerous risks associated with food. Food risks, in fact, have a special standing in people's risk appraisals (Knox, 2000). Not surprisingly these concerns are shared by experts, public policy makers, and officials of health-related organizations (Payson, 1994). Indeed, healthcare cost due to illness associated with food hazards, food poisoning, and aberrant food intake have steadily increased over the past 20 years (cf. Brennan et al., 2007).

Despite the importance of the issues and the relevance of the research on food processing, comprehensive and validated databases of stimuli are rather limited, outdated, or not available for non-commercial purposes to independent researchers who aim at developing their own research program. The FoodCast research image database (FRIDa) is an attempt to fill this gap by providing the scientific community with a flexible stimulus-set, validated on a sample of young healthy individuals and that could be used for neuroscientific investigations.

Food perception is routed in multiple sensory modalities (see Rolls, 2005, for a review). Nevertheless, an increasing number of studies have been focusing on the visual processing of food (i.e., images of food; e.g., Killgore et al., 2003; Simmons et al., 2005; Toepel et al., 2009; Frank et al., 2010; Nummenmaa et al., 2012). This approach seems very appropriate to experimental investigations of the neural substrates of decision making about food as it resembles a large number of real life situations (e.g., food purchase, food choice, etc.).

Recent functional neuroimaging studies have provided insights as to how the human brain processes different aspects of food. The brain network involved in food perception includes projections from the insula to the orbitofrontal cortex (OFC) and the amygdala, which are reciprocally connected and both project to the striatum (Rolls, 2005), while other frontal regions, such as the dorsolateral prefrontal cortex, are involved in decision making about food (McClure et al., 2004). Importantly, these regions are active both when tasting and smelling food samples (O'Doherty et al., 2001; Gottfried and Dolan, 2003) and during visual processing of food (Killgore et al., 2003; Simmons et al., 2005; Frank et al., 2010; Stingl et al., 2010; Nummenmaa et al., 2012).

A key factor modulating brain activations during exposure to food seems to be the energy content of the viewed food. High-calorie content activates the OFC to a greater extent compared to low-calorie content food (Frank et al., 2010), as well as the prefrontal cortex, diencephalon (Killgore et al., 2003), and primary gustatory cortex (i.e., the insula; Simmons et al., 2005; Nummenmaa et al., 2012). Despite the growing number of studies reporting brain activations associated with different aspects of food, the approach taken so far has not always distinguished between different types of food. One exception is represented by research on dieting behaviors and regulation where categories such as “attractive/palatable food” and “neutral/control food” are sometimes compared, though without a clear validation procedure that considers important variables for food choice (e.g., Papies et al., 2008). Instead, little is known as to whether our brain distinguishes between food categories and how eventually does so.

In general, neuropsychological and neuroimaging studies have suggested that concepts may be represented in different categories depending on whether or not they are natural entities or artifacts (see Forde and Humphreys, 2001, for a review; Mahon and Caramazza, 2009). Some patients have been described as having a selective deficit for recognizing natural but not artificial objects (e.g., Warrington and Shallice, 1984) and *vice versa* (e.g., Warrington and McCarthy, 1983, 1987). Several principles have been proposed that could be responsible for this conceptual organization. According to the sensory and functional/motor theory, the recognition of living things relies more on perceptual features (e.g., shape, texture, color, sound, etc.), while the recognition of non-living things relies more upon their functions and the actions that they allow (Warrington and Shallice, 1984). In contrast, Caramazza and collaborators argued that conceptual knowledge is organized according to domain-specific constraints and suggested that “there are innately dedicated neural circuits for the efficient processing of a limited number of evolutionarily motivated domains of knowledge” (p. 97, Mahon and Caramazza, 2011; see also Caramazza and Shelton, 1998; Caramazza and Mahon, 2003).

None of these theories, however, provides a satisfactory account for how food should be processed by the brain. Food is normally processed as a multisensory stimulus (i.e., sight, smell, taste, and touch) and therefore shares the properties of living things from which it originates. Consequently, in patients with selective deficits for natural objects, the difficulties seem to extend also to the domain of food (Borgo and Shallice, 2003). Food, however, has also functional characteristics and, like tools, is also the product of human labor with specific functions and effects on our body (e.g., diuretic, refreshing, etc.). This functional information about food is spontaneously used by humans, as demonstrated by research in which both healthy subjects and patients with eating disorders classify food according to its function (Urdapilleta et al., 2005). Following this line of reasoning, some foods should be processed by our brain as a handmade/tool-like object.

Food is often a compound of different edible ingredients (e.g., chicken croquettes are made of chicken, egg, breadcrumbs, etc.) raising the question of whether humans understand processed food as single natural entities or as handmade compounds. It is important to understand how we process food differently in its original form compared to after being transformed. Not surprisingly, some scholars attribute a fundamental importance in human evolution to the advances in food preparation (Wrangham, 2009). Here we consider a food as transformed if the transformation applied by humans has changed the food's organoleptic state. This definition includes cooking (e.g., boiled zucchini), aggregation (e.g., mayonnaise), and preservation processes (e.g., cured meat or pickling). Based on this definition, items such as dry parsley, dates, and raisins are considered natural food (because the natural dehydration occurs without human intervention as, instead, is necessary when salt or sucrose is added); on the other hand, items such as preserved olives are considered transformed food.

With *FRIDa* we first aimed at providing a database of stimuli where important variables are taken into account. Among the most relevant variables, we considered caloric content (both actual and perceived), food transformation category (natural food vs. transformed food), perceived level of transformation (on the continuum from natural to highly transformed food), and the type of food (vegetable, meat, starch, etc.). In addition to these “food-specific” variables, in validating FRIDa we considered other variables such as valence, arousal, familiarity, and typicality. Finally, we also provide data on the visual features of the stimuli, such as visual ambiguity, size, brightness, and high spatial frequency power. According to Knebel and colleagues (2008), these variables need to be well controlled particularly in imaging experiments.

Our second goal was to provide a large number of items from categories other than food that could serve as control stimuli for different relevant aspects. For instance, one important decision that humans have to make is to distinguish what is edible (e.g., fruits and vegetable) from what is not (e.g., tree and leaves). In this respect, FRIDa offers a set of photographs of natural objects that have sensorial representations, like food, but are not edible. A second example of a control category included in FRIDa is rotten food, that is food no longer edible.

Taken together, FRIDa comprises a large set of images of food and non-food items, with food images including natural-food (e.g., strawberry and pepper), transformed-food (e.g., french fries and baci di dama), and rotten-food (i.e., uneatable; e.g., rotten banana). Non-food images include inedible natural items (e.g., pinecone), artificial food-related objects (e.g., teacup and pizza cutter), artificial objects (e.g., guitar and gloves), animals (e.g., camel), and interior and external scenes (e.g., airport and theater).

A validation procedure of all stimuli was performed as detailed in the next section. Finally, we report the results of the analyses aimed at understanding how the variables we considered interact with each other.

## Methods

### Participants

Eighty-six native-Italian speakers (48 females) took part in the validation experiment. Participants were recruited via an advertisement posted on a dedicated social-networking site, and were monetarily rewarded (€15) for their participation. Summary of the participants' demographic information is reported in the “Results” section.

#### Participants' exclusion criteria

As we aimed at providing a database validated on a general population of young adults without eating disorders, pathological conditions, or altered physiological states, we excluded participants who showed signs of aberrant eating behavior/patterns and/or behavioral symptoms commonly associated with risks of eating disorders on the *Eating Disorder Inventory-3* (*EDI-3;* Garner et al., 1983), those who acknowledged to consume neurotropic substances, those who had dietary restrictions for medical or religious reasons, and those who reported having fasted for more than 5 h prior to the experiment and reported being extremely tired, hungry, thirsty. In addition, to increase the representativeness of the reported data for the population of reference, here we will only report the data relevant to participants with an age range of 18–30 and with a Body Mass Index (BMI) within the range of 17–27. This sample well represents the range of healthy young people typically taking part of research in psychological sciences.

### Stimuli

**Figure 1:**
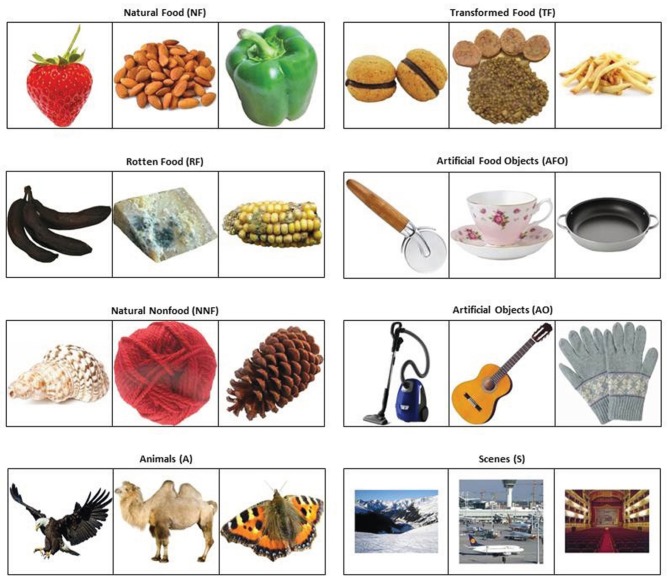
**Examples of the stimuli from the database.** Tree examples of items from each category of stimuli. Information regarding the examples is reported in the Appendix.

The final database comprises 877 images. All images are open-source and compiled from a web-based search. Each image depicts an item belonging to one of eight different categories: (1) natural-food (e.g., strawberry; *N* = 99 images); (2) transformed-food (e.g., french fries; *N* = 153 images); (3) rotten-food (e.g., moldy banana; *N* = 43 images); (4) natural-non-food items (e.g., pinecone; *N* = 53 images), (5) artificial food-related objects (e.g., teacup; *N* = 119 images); (6) artificial objects (e.g., guitar; *N* = 299 images); (7) animals (e.g., camel; *N* = 54 images); and (8) scenes (e.g., airport; *N* = 57 images). Figure 1 displays three examples of picture for each category.

The pictures depicted different amounts of the food including a small portion of food (e.g., one cherry, one mushroom, and one cookie; *N* = 30), a standard portion size (e.g., one tomatoes, one apple, and a main dish; *N* = 137), and large portions (one whole pineapple, one whole cake, and a whole roasted kitchen; *N* = 85). In doing so, we provided a wide array of different food items. Moreover, we aimed at obtaining food stimuli of comparable visual sizes. This result would be difficult to achieve if represented portions were matched, e.g., if pictures always showed two specimens of fruit, meat cuts, and so on.

All images are color photographs, with a minimum resolution of 600 × 600 pixels. All texts and symbols were removed. The item represented in each photograph was first cropped and then placed on a white background. In order to achieve uniformity on visual features, the open source software IrfanView 4.30 (http://www.irfanview.com/) and custom-written MATLAB® codes (Mathworks, Natick, Massachussets, USA) were used. In order to achieve comparability of visual features across categories, scenes (which cannot be cropped because they lack a principal subject) have been wrapped in a white background. Images were then resized to a standard dimension of 530 × 530 pixels, converted to an RGB-color format, and saved as bitmap images (“.bmp” extension).

### Visual features extraction

For each image, we extracted stimulus size, mean brightness, and high spatial frequency power. Since all items were shown on a white background, stimulus size was defined as the ratio between non-white pixels and total image size. Given that all images were resized to 530 × 530 pixels, the number of pixels covered by the stimulus can be readily computed by multiplying this ratio by 530 × 530. To evaluate brightness, we computed the mean brightness of the grayscale version of the image. To calculate spatial frequency we employed a bi-dimensional fast Fourier transform^1^, which converts the image represented as an array of brightness values to its unique representation in the spatial frequency domain. After transformation, individual pixels represent the power of specific spatial frequencies in a given direction.

### Validation procedure

Upon arrival, participants signed a written informed consent. Participants were then seated in front of a desktop computer where they could self-administer the experiment. Standardized instructions presented on the screen described each phase and each task of the experiment. The whole experimental session lasted approximately 1.5 h. After collecting the demographic information (demographic phase), participants began to perform the proper experiment that consisted of a naming task followed by a rating task (rating phase).

#### Demographic phase

After having reported their age, gender, weight, height, and handedness, participants answered five questions regarding their current psycho-physical state (in random order). Participants responded by clicking with the mouse on the appropriate point of a continuum. Responses were analyzed by converting distances to a scale ranging from 0 to 100, although this was not explicitly displayed to the participants. The five questions were the following:

(a) “How hungry are you now?” (With the two extremes labeled: 0 “not at all hungry” and 100 “very hungry”).
(b) “How thirsty are you?” (With the two extremes labeled: 0 “not at all thirsty” and 100 “very thirsty”).
(c) “How much time did pass since your last complete meal?” (With the two extremes labeled: 0 “less than an hour” and 100 “more than 5 hours”).
(d) “How much time did pass since you last have eaten something?” (With the two extremes labeled: 0 “less than an hour” and 100 “more than 5 hours”).
(e) “How tired are you now?” (With the two extremes labeled: 0 “not at all tired” and 100 “very tired”).

At the end of the experiment, the participants were also administered the *EDI-3* symptoms checklist questionnaire (Garner et al., 1983), normally used for evaluating symptoms commonly associated with Anorexia Nervosa e Bulimia, with the aim of identifying any participant that could eventually be at risk of eating disorders or with aberrant eating behaviors/patterns. The EDI-3 covers topics related to the management of diet and of personal habits related to weight control. The questionnaire includes eight different topics: (1) dieting habits, (2) physical exercise, (3) binge eating, (4) vomiting, (5) laxatives, (6) diet pills, (7) diuretics, and (8) relevant medications. Within each of these areas, the questions investigate the onset of a behavior (e.g., When did you induce vomit for the first time?), its frequency, its last occurrence, and its persistence. Participants that are not at risk of eating disorders generally show no symptoms and no risky behaviors related to these eight areas. Sometimes even though a participant is not at risk of eating disorders he/she may still show some relevant behaviors in one of the areas (e.g., the presence of binge eating only). Based on these considerations we excluded participants if they showed: (a) an impairment in three or more of the eight areas, (b) deviant behaviors (e.g., induction of vomiting) in the 3-month period preceding the administration of the questionnaire, and (c) reported a high frequency of occurrence of the abnormal behavior.

In addition to the EDI-3, participants were also asked to complete a second set of questions concerning the personal preferences and dietary restrictions; they had first the opportunity to classify themselves as omnivore, vegetarian, or vegan, and then they were asked to indicate any dietary limitations (both in food choice, preparation requirements, and fasting) based on religious beliefs and/or medical reasons.

#### Written naming task

Participants were presented with one item at a time, and asked to type its name into a dialog box. Each photograph was displayed until a response was provided. The goal of this task was to collect the most commonly-used linguistic referent for each of the items. Given the expansiveness of the database, participants were presented with only a subset (175 images) of randomly selected images from each image category. Thus, each participant judged a different subset of the images. For this reason each image was rated by a different, variably overlapping subsample of raters. However, the assignment of each picture to the subset of raters was done randomly, successfully reducing the risk of unwanted effects by non-measured variables that may differentiate the distinct subsample of raters. This procedure is commonly used in validation procedures for databases (e.g., BEAST database; deGelder and VandenStock, 2011; GAPED database, Dan-Glauser and Scherer, 2011; IAPs databases; Lang et al., 2008).

#### Rating phase

This phase included eight different blocks in each of which participants performed a different judgment. After each block, participants took a short break. Again, for each judgment participants were presented with only a subset of images from the original database randomly selected for each participant. The only restriction was that the number of images presented to each participant for each category of image (i.e., natural-food, transformed-food, etc.) was proportionate to the total number of images in each category to be validated (i.e., each participants saw three times more artificial objects than natural-food since the former are 299 in total while the latter are 99 in total).

The order of the rating was as following. During the rating phase, first participants rated only food items on the three variables (perceived calorie content, perceived distance from eatability, perceived level of transformation; in random order); then participants rated the items from all the image-categories on five other dimensions (valence, arousal, familiarity, typicality, ambiguity; in random order). For each judgment, each stimulus picture was presented one at a time together with a continuous scale underneath it. Participants expressed their judgment by clicking with the mouse on the appropriate point of a continuum. Responses were analyzed by converting distances to a scale ranging from 0 to 100, although this was not explicitly displayed to the participants. According to the type of judgment, the extremes of the scale were differently labeled (see below).

The rating phase consisted on the subjective rating relative to the following dimensions rated in different blocks:

(a) *Perceived calorie content* of the item (tested on food images only). Exact “calorie content” of the represented food is based on published measures (Marletta et al., 2000), but this rating provides an index of perceived energy density. Although this estimate correlates with fat content (see Toepel et al., 2009), it is, however, important to distinguish between the two because in some images calorie content maybe more difficult to be inferred. The question we asked was: “how much calorie content do 100 g of the food represented in the picture provide?” The extremes of the scale were labeled as “low calorie content” (0) and “high calorie content” (100).
(b) *Perceived distance from eatability* (asked about food images only). “Distance from eatability” is a measure of the work still required to bring the depicted food item into an edible form (e.g., raw meat will generally require more work than fruit). The instructions for the participants about the task provided a few examples of food items (not included in the database) to explain the rationale and the meaning of the question. The question during the rating phase was: “how much work is necessary to bring the food represented in the image ready to eat?” The extremes of the scale were labeled as “very little work” (0) and “a lot of work” (100).
(c) *Perceived level of transformation* (tested on food images only). “Transformation” implies a judgment on the amount of processing a food underwent (a cake has a higher level of transformation than potato chips because it requires more elaborate processing to obtain it from its starting ingredients). The instructions for the participants about the task provided a few examples of food items (not included in the database) to explain the rationale and the meaning of the question. In particular, the instructions explained that participants should consider only the work necessary from the initial ingredients. For instance, in judging how much work is required to prepare an apple cake, participants should consider the amount of elaboration that goes into combining the starting ingredients (apples, milk, butter, etc.) without considering the amount of work to cultivate an apple tree and to grow cows in order to obtain milk and butter. The question was: “how much work was required to prepare the food represented in the image?” The extremes of the scale were labeled as “no work at all” (0) and “a lot of work” (100).
(d) *Valence* of the item (tested on all image categories). “Valence” expresses the pleasantness of the image. The question was: “how negative/positive is the item represented in the image?” The extremes of the scale were labeled as “very negative” (0) and “very positive” (100).
(e) *Arousal* experienced viewing the picture (tested on all image categories). “Arousal” indexes the emotional salience of the item (i.e., how aroused a person reports to be while viewing the image). The question was: “how arousing is the presented image?” The extremes of the scale were labeled as “not at all” (0) and “extremely” (100).
(f) *Familiarity* of the item (tested on all image categories). “Familiarity” refers to the frequency with which a person reports encountering the item in his/her daily life. The question was: “how often do you encounter what is represented in the picture in your daily life?” The extremes of the scale were labeled as “never” (0) and “often” (100).
(g) *Typicality* of the image (tested on all image categories). “Typicality” requires a judgment of how typical the item depicted appears for its category. The question was: “how typical is what is represented in the image of its category?” The extremes of the scale were labeled as “not at all” (0) and “very much” (100).
(h) *Ambiguity* of the image (tested on all image categories). “Ambiguity” requires a judgment of how easy it is to correctly identify the subject of the image. The question was: “how easy/difficult is to understand what is represented in the image?” The extremes of the scale were labeled as “very easy” (0) and “very difficult” (100).

Because of their different speed in performing the judgment tasks, not all participants completed the task and, thus, expressed the same number of judgments. On average participants expressed 1390 rating judgments over the eight rating tasks.

## Results

FRIDa with the relative validation data can be obtained for non-commercial research use upon request (http://foodcast.sissa.it/neuroscience/).

### Demographic information

Based on the responses on the EDI-3, three participants were excluded from the original sample as they reported abnormal behaviors related to binge eating, vomiting, the use of laxatives, diuretics, diet pills, and the use of drugs to lose weight. We also excluded participants who reported adherence to dietary restrictions for religious beliefs (*N* = 1) and/or for medical reasons (*N* = 2). We additionally excluded participants who had an age outside the target age range of 18–30 (*N* = 4). Finally, we excluded participants who had a BMI outside the range of 17–27 (*N* = 3).

Thus, the final sample included 73 participants (39 females; 64 right handed). The final sample's average age was 23.1 years (*SD* = 3.3; *Range* = 18–30). Participants, whose weight and height were used to compute participant's BMI (BMI = kg/m^2^), which is a heuristic proxy for human-body fat even though it does not actually measure the percentage of body fat, were within the range of non-problematic body-mass index with an average BMI of 21.6 (*SD* = 2.4; *Range* = 17.30–26.83; Female *Mean* = 20.6; *SD* = 2.2; *Range* = 17.3–26.6; Male *Mean* = 22.7; *SD* = 2.2; *Range* = 17.9–26.8).

Of the 73 participants included in the final sample, 3 participants described themselves as vegetarian and 1 as vegan. Few participants (*N* = 4) reported some form of mild sensitivity that, however, were not the product of strict dietary restrictions.

### Participants' psycho-physical state

On the 0–100 scale, participants responded to five different questions. When asked to rate how hungry, thirsty and tired they were, participants reported, respectively, a low hunger level (*Mean* = 20.4, *SD* = 24.9), low level of thirst (*Mean* = 35.3, *SD* = 23.3), and low level of tiredness (*Mean* = 30.6, *SD* = 23.7). In addition, participants were also asked to report the amount of time passed from the last full meal and the last snack. Participants reported to have had, on average, the last full meal approximately 2.5 h prior to the experiment (*Mean* = 54.2, *SD* = 39.4) and that they had their last snack approximately an hour a half before the experiment (*Mean* = 27.1, *SD* = 29.1). Based on the self-assessed hunger level and the fact that no participant took part in the experiment after having fasted completely for a long time, we can conclude that participants were not in a physiological state (extremely hungry or after a long fasting time) that could potentially affect their rating.

### Validation ratings

One goal in developing FRIDa was to create a flexible tool by providing a large number of images in each category. In this way, researchers can choose a targeted subset of stimuli depending on the objectives of the experiment they wish to run. Based on the validation data, in fact, researchers can control, equate or manipulate the different variables in a given experiment by selecting the appropriate subset of images. Within this framework, analyses on the aggregated data may not be so informative since the different categories comprise a large number of images and because for any variable the within category variation may be quite high. However, below we report a short summary of the main variables by image category (see also Table 1) and the correlations for three separate subsets of images (Tables 2–4).

**Table 1:** **Average ratings aggregated for each food category (standard deviations are in parentheses)**.

	**Ratings**							
	**Valence**	**Familiarity**	**Typicality**	**Ambiguity**	**Arousal**	**Perceived calorie content**	**Distance from eatability**	**Level of transformation**
Natural-food	65.55 (9.49)	58.32 (18.94)	76.54 (11.07)	10.76 (9.16)	31.46 (11.46)	20.76 (14.84)	29.92 (19.92)	6.43 (7.15)
Transformed-food	65.317 (9.22)	53.28 (14.43)	68.46 (13.14)	16.98 (12.09)	48.87 (11.73)	67.79 (14.65)	14.64 (10.26)	58.45 (15.03)
Rotten food	6.58 (4.44)	12.44 (7.46)	13.08 (5.50)	43.21 (21.79)	25.56 (8.10)	22.12 (12.84)	57.93 (8.10)	13.67 (12.96)
Natural non-food items	59.88 (15.58)	33.98 (20.75)	63.07 (13.57)	18.02 (13.57)	37.02 (12.79)			
Artificial food-related objects	58.75 (6.94)	54.32 (24.17)	66.15 (15.71)	11.76 (11.77)	20.88 (8.18)			
Artificial objects	57.95 (10.52)	45.09 (25.89)	70.30 (12.87)	8.09 (8.03)	26.98 (13.90)			
Animals	54.20 (17.21)	18.62 (20.97)	63.85 (10.46)	12.51 (8.22)	50.13 (12.46)			
Scenes	60.18 (15.84)	33.94 (22.74)	66.35 (10.67)	14.18 (6.99)	46.29 (17.67)			

**Table 2:** **Correlation between validation dimensions for food (natural-food and transformed food, *N* = 252)**.

	**Ratings type**							
	**Valence**	**Familiarity**	**Typicality**	**Ambiguity**	**Arousal**	**Perceived calorie content**	**Distance from eatability**	**Level of transformation**
Valence		0.55^**^	0.46^**^	−0.38^**^	0.44^**^	0.01	−0.36^**^	0.08
Familiarity			0.68^**^	−0.52^**^	0.04	−0.24^**^	−0.22^**^	−0.19^*^
Typicality				−0.73^**^	0.02	−0.26^**^	−0.04	−0.36^**^
Ambiguity					−0.01	0.24^**^	0.02	0.35^**^
Arousal						0.66^**^	−0.43^**^	0.66^**^
Perceived calorie content							−0.38^**^	0.88^**^
Distance from etability								−0.43^**^
Level of transformation								

* Correlation is significant at the 0.05 level (2-tailed).

** Correlation is significant at the 0.01 level (2-tailed).

**Table 3:** **Correlation between the validation dimensions for objects (artificial food-related objects and artificial objects, *N* = 418)**.

	**Ratings type**				
	**Valence**	**Familiarity**	**Typicality**	**Ambiguity**	**Arousal**
Valence		0.35^**^	0.80^**^	−0.60^**^	0.59^**^
Familiarity			0.50^**^	−0.31^**^	−0.05
Typicality				−0.74^**^	0.43^**^
Ambiguity					−0.39^**^
Arousal					

** Correlation is significant at the 0.01 level (2-tailed).

**Table 4:** **Correlation between validation dimensions for natural items (rotten-food, natural-non-food item, animals, and scenes, *N* = 207)**.

	**Ratings type**				
	**Valence**	**Familiarity**	**Typicality**	**Ambiguity**	**Arousal**
Valence		0.30^**^	0.24^**^	−0.28^**^	0.35^**^
Familiarity			0.53^**^	−0.35^**^	−0.10^*^
Typicality				−0.59^**^	0.08
Ambiguity					−0.20^**^
Arousal					

* Correlation is significant at the 0.05 level (2-tailed).

** Correlation is significant at the 0.01 level (2-tailed).

Irrespective of the participant expressing them, the ratings (for each judgment) on a specific item were averaged (e.g., all the ratings on familiarity for the item “Lion” were aggregated). Each item score was then averaged together with the score of the other items of the same category to obtain the summary data reported in Table 1 (e.g., familiarity score for the category animals is the average of the average familiarity scores relative to all the items belonging to this category). Table 1 summarizes the aggregated validation data by image category and by types of judgment. Table A1 provides the overview of the validation information available for any given picture in FRIDa and the data for three exemplars of each image category (the data are available upon request from the authors together with the pictures database). In Table A2 one can find the validation ratings, split by gender, for three exemplars of each image category. This information is available in FRIDa for any given picture (see Tables A1, A2). In the following we report independent samples *T*-test comparing natural-food and trasformed-food. Degrees of freedom were adjusted (if necessary) after Levene's test for equality of variances.

(a) *Perceived calorie content* Natural-food was perceived as containing, in general, fewer calories (*Mean* = 20.76, *SD* = 14.84) than transformed-food (*Mean* = 67.79, *SD* = 15.39; *t*_(250)_ = 23.98, *p* < 0.001).
(b) *Perceived distance from eatability* Natural-food required more work (*Mean* = 29.92, *SD* = 19.92) than transformed-food (*Mean* = 14.65, *SD* = 10.26; *t*_(130)_ = 7.22, *p* < 0.001) supporting the idea that transformed-food is generally perceived as more ready to be eaten.
(c) *Perceived level of transformation* Natural-food was perceived as less processed (*Mean* = 7.43, *SD* = 7.15) than transformed-food (*Mean* = 58.45, *SD* = 15.03; *t*_(234)_ = 36.88, *p* < 0.001).
(d) *Valence* Importantly, natural-food (*Mean* = 65.55, *SD* = 9.49) and transformed-food (*Mean* = 65.31, *SD* = 9.22) were both positive in valence, and did not significantly differ from one another, *t*_(250)_ = 0.84, *ns*. It can be noted that, not surprisingly, the remaining categories were all slightly above midpoint with the exception of rotten-food that was considered very negative (*Mean* = 6.58, *SD* = 4.44).
(e) *Arousal* The eight categories showed a large variation in arousal (see Table 1).
(f) *Familiarity* Natural-food items (*Mean* = 58.32, *SD* = 18.94) were slightly more familiar than transformed-food items (*Mean* = 53.28, *SD* = 14.43), *t*_(167)_ = 2.25, *p* < 0.05. The other categories showed different levels of familiarity.
(g) *Typicality* In general, all the categories showed a high level of typicality demonstrating that the pictures chosen were good examples of the relevant objects and were typical of their respective categories (see Table 1).
(h) *Ambiguity of the images* For all of the categories, the images showed a very low level of ambiguity as the images were rated as very easy to be identified. The only exception in ambiguity was found in the category “rotten-food” (*Mean* = 43.21, *SD* = 21.79). However, this is expected as the process of degeneration tends to transform and “disguise” the features of the original food with molds, dehydration, etc.

### Correlational analyses

Since many variables have been taken into account in FRIDa, users should be aware of co-variation between them when deciding on the criteria for selecting the images. Based on the ratings, we computed Pearson bivariate correlations separately for the three aggregated subsets of images: (a) food (natural-food and transformed-food; see Table 2), objects (artificial food-related objects and artificial objects; see Table 3), and natural items (rotten food^2^, natural-non-food, animals, and scenes; see Table 4).

In general, based on the variables rated for all item categories (i.e., valence, familiarity, typicality, ambiguity, and arousal), the three image subsets (food, objects, and natural items) showed parallel correlation results with only some magnitude differences. In all three subsets, in fact, we found that valence ratings positively correlates with familiarity (Pearson *r*-range = 0.30/0.55), typicality (*r*-range = 0.24/0.80), arousal (*r*-range = 0.35/0.59), and correlate negatively with ambiguity (*r*-range = −28/−0.60). That is to say, the more positive an item tends to be rated, the more familiar, typical and arousing and the less ambiguous it is perceived. In addition, ambiguity is also negatively correlated with familiarity (*r*-range = −0.31/−0.52) and typicality (*r-range = −0.58/−0.74*): this means that the more ambiguous an item is perceived, the less familiar and less typical it is rated.

The three image groups (food; objects, and natural items), however, show also some interesting differences about the correlations in which arousal is involved. While both objects and natural items are rated more arousing when less ambiguous (*r* = −0.20, −0.39, respectively), objects are rated more arousing when less familiar (*r* = −0.10); natural items, instead, are considered more arousing when more typical (*r* = 0.43). These results are not surprising, as arousal depends more on the clear identification of the arousing item; however, these results do not extend to food items. In fact, the level of arousal induced by food items is not correlated with familiarity, typicality, and ambiguity.

Moreover, the correlation of brightness with arousal and valence was computed, while controlling for other low-level visual features of the images such as size (since size determines the quantity of the white portion in the picture and this affects brightness), and spatial frequency power. We found that in the database, brightness is negatively correlated with arousal for natural items (*r* = −0.16, *p* = 0.019), marginally correlated for objects (*r* = −0.08, *p* = 0.09), and it is not correlated for food (*r* = −0.03, *ns*.); on the other hand, brightness is only marginally negatively correlated with valence for natural items (*r* = −0.12, *p* = 0.08), and did not correlate with it for food (*r* = 0.06, *ns*.) nor for objects (*r* = −0.02, *ns*.).

In addition to these variables, however, food items were also rated for perceived calorie contents, perceived distance from eatability, and level of transformation (see Table 2). For food, arousal ratings correlate positively with perceived calorie content (*r* = 0.66) and level of transformation (*r* = 0.66), while they correlate negatively with distance from eatability (*i* = −0.43). Thus, food items tend to be rated more arousing when are judged to contain more calories, when they are more transformed and when they require less work in order to be eaten. It is noteworthy that the level of arousal seems to be in some way connected to the desire to immediately consume a food item. This is also supported by the negative correlation between valence and distance from eatability (*r* = −0.36) suggesting that the less work is required in order to eat an item, the more positively the item is rated. Perceived calorie content also correlated with level of transformation (*r* = 0.88) and distance from eatability (*r* = −0.38), and the latter two also correlated with each other (*r* = −0.43). Finally, perceived calorie content also positively correlated with the actual calorie content (*r* = 0.73, *p* < 0.001).

As participants somewhat differed for BMI and age, a correlational analysis was performed to assess the potential effect of these differences on the ratings. In addition, also the number of female and male raters (assigned randomly to each picture) may vary from picture to picture and was also analyzed. When analyzing the effect of these variables on the ratings of each picture (controlling for the objective characteristic of the pictures such as brightness, high frequency, size and frequency in language) there were no systematic effects of BMI, age or proportion of female rater. The only exception was that the older the raters, the more typical tended to be rated the images (*r*_(870)_ = 0.13, *p* = 0.001). This effect persisted when smaller range of age was considered (20–30). Moreover, the percentage of female raters in the subset seemed to be negatively correlated with arousal (*r*_(870)_ = −0.10, *p* = 0.01).

The FRIDa database also contains data for each of the images for which we are not reporting aggregated analyses, but that can be easily derived by researchers who are interested.

## Discussion

Here we presented FRIDa, a database of food and non-food items. Our first aim was to develop and validate a large database of images that could be freely available to a wide range of researchers. We validated 877 colored images with a final sample of 73 healthy participants. In the validation procedure, we included standard variables (i.e., valance, arousal, familiarity, calorie content, and typicality) which have been taken into account also in previous studies on visual processing of food items (Toepel et al., 2009; Frank et al., 2010; Nummenmaa et al., 2012; Pergola et al., 2012).

The aggregated results of the validation procedure support the orderliness of the data, in that we observed high inter-variable correlations among the standard variables. The present database allows for the selection of a subset of stimuli set from two or more categories according to the desired criterion, as shown in previous research (Pergola et al., 2012). The correlation analyses clearly show that food is a special category compared to natural items (rotten-food, animals, natural-non-food items, and scenes) and objects (artificial food-related object and artificial objects). While for objects, “novelty” seems to be related to arousal, for natural items the typicality of an item is related to its arousal level (e.g., the more typical image of a lion will induce higher levels of arousal). Instead, arousal ratings on food fail to show such correlation pattern. In fact, apart from the positive correlation with valence, arousal ratings on food items show no significant correlation with familiarity, typicality, or ambiguity.

In the present study, we have also included other variables related to food items (e.g., food transformation category, perceived level of transformation rating, and food taxonomy) that, to our knowledge, have never been considered in the extant research on food. As discussed above, we expect that these variables will play a role in food processing. In line with this reasoning, FRIDa also included data on the visual features of the stimuli, such as visual ambiguity, size, brightness, and high frequency power. Importantly, size was evaluated with an objective and replicable method, whereas in previous works its evaluation relied on visual inspection (Stingl et al., 2010).

In research on food processing, calorie content is an important variable (Killgore et al., 2003; Toepel et al., 2009; Frank et al., 2010; Nummenmaa et al., 2012). Toepel et al. (2009), for instance, reported a positive correlation between perceived and actual fat content. In FRIDa, in which both perceived and actual calorie content are included, this correlation was replicated. However, it is indeed useful to have both kinds of information in order to evaluate separately subjects' expectations in terms of energetic value and actual energetic content. This does not apply only to fatty food, but also to food in which calories are primarily dependent on glucides and proteins (e.g., natural-food).

We placed a great interest in possible differences between transformed food and natural food. While they do not differ in valence and familiarity, these two types of food show important differences in arousal, in perceived calorie content, and in distance from eatability, suggesting that this is an important distinction that should be considered in research on food perception. Rudenga and Small (2012) have recently shown that activity in the amygdala after ingestion of sucrose solutions is modulated by consumption of artificial sweeteners. Artificial sweeteners are a rather extreme example of transformed food, and the level of transformation may critically affect brain correlates of food perception. Wrangham (2009) goes as far as attributing a fundamental importance in human evolution to the advances in food preparation. At least in rats, it has been shown that food preparation affects the energy that can be extracted during digestion. The same food, in fact, may yield different calorie content depending on the way it has been prepared since the organism may need different amounts of energy to metabolize it (Carmody et al., 2011). For this reason, the degree to which a food item has been transformed has a biological salience, not to mention the cultural importance of food preparation in human societies (Wrangham, 2009) and the possible implications for choice and behavior as calorie content has been linked to subsequent taste judgments and unrelated cognitive tasks (Harrar et al., 2011; Ohla et al., 2012).

Thus, there are reasons to believe that humans mentally process and treat food differently when it is in its original, “natural” form compared to when it has been transformed. Current evidence suggests that the level of transformation covaries with calorie content and arousal, which immediately hints to an alternative explanation of the different brain activation patterns found when participants viewed low- vs. high-calorie content food (Killgore et al., 2003; Toepel et al., 2009; Frank et al., 2010; Nummenmaa et al., 2012). Food preparation provides metabolic advantages for digestion since transformed food requires less effort to be digested. Moreover, normally food preparation enhances the calorie content of food by adding highly energetic ingredients (typically, fats) that might be difficult to track based on visual cues. For example, an important portion of the energy that can possibly be extracted from a salad depends on the dressing.

Even though the current data indicates that subjects are aware of these differences, the work by Rudenga and Small (2012) suggests that natural and transformed foods may be differently evaluated and hence decision-making about food could be affected by this component. A very telling example of the potential relevance of this dimension for our understanding of how humans process food-related information comes from our near past. Recently, there was great concern about the transmission of the H5N1 virus (“bird flu”) from birds to other animals and, in particular, to humans. People drastically reduced the consumption of chicken meat worldwide, even though there was no clear evidence that the virus could be transmitted in this way. Surprisingly, the consumption of food *containing* chicken, such as chicken croquettes, decreased to a much lesser extent (Beach et al., 2008). FRIDa will hopefully open the way to studies that control for this variable, which so far has been considered only by *excluding specific food categories* from the stimulus material (see for example Simmons et al., 2005).

One of the main aims we pursued in constructing FRIDa was to provide the same validation data also for non-food items that could be employed as controls in studies on food processing. FRIDa provides access to several types of control conditions. The natural non-food items are examples of items consisting of the same chemical compounds characterizing food but are nevertheless inedible. On the other hand, objects are available in FRIDa with and without a semantic connection to food (artificial food-related objects and artificial objects). Additionally, we provided pictures of rotten food, which consist of the same chemical compounds as food items, are related to food, can provide energy, and yet are uneatable. For comparability with classical neuropsychological and neuroimaging research, we also screened pictures of animals and scenes (Borgo and Shallice, 2003; Simmons et al., 2005). These additional categories will allow for the investigation of many different aspects of food processing as well as behavior related to food. Notably, the non-food items can also be used in their own right. FRIDa could well be used in research on memory, as it offers a great deal of items from different categories for which a lot of relevant information is provided (e.g., word frequency, name agreement) as well as research on cognitive processes more generally.

The present validation provides comprehensive information on each one of 877 pictures. Seventy-three healthy normal-weight young participants took part in the study from which we excluded three who showed clinical symptoms of eating behavior disorders. Additionally, we excluded participants who followed dietary restrictions for religious belief or medical conditions but we did not screen for food preferences: the possible variation of dietary preferences shows that our sample of participants is representative of the general young population with respect to healthy dietary habits. FRIDa provides validation data for a sample of both male and female participants where each image has been rated by at least 16 raters (*M* = 18.86; *SD* = 0.96; *Range* = 16–25). Previous research has shown that variables such as gender and BMI may have an impact on food processing (Toepel et al., 2012). For the present research, we therefore limited our sample to those participants with a BMI between 17 and 27. Since food studies are relevant for both females and males, we included both genders, and we provided the validation data for the full sample as well as separately for female and male raters. To make FRIDa as flexible and useful as possible, data relative to subsamples of participants according to specific selection criteria (e.g., age 18–24) can be obtained from the authors upon request.

In conclusion, FRIDa is a freely available and flexible database of food and non-food items (877 colored images) that can be used by a wide range of researchers. The validation data are derived from a sample of 73 participants with an age range of 18–30 with no dietary restrictions for medical or religious reasons nor aberrant eating behaviors. The sample we used represents the population that normally takes part in experiments performed in psychological studies. Future efforts should, however, be directed at enriching the database by collecting validation data also from other samples (e.g., children, elderly, and patients with eating disorders). Finally, in this validation experiment, a few variables such as the menstrual cycle and smoking habits, that may be of interest in relation to food processing and food choices, have not been investigated and future research should also consider filling this gap.

### Conflict of interest statement

The authors declare that the research was conducted in the absence of any commercial or financial relationships that could be construed as a potential conflict of interest.

## Footnotes

^1^The power spectrum was normalized by dividing the absolute value of the coefficients associated to different frequencies by the size of the images. A second normalization step rescaled power in the range [0,1]. A third step multiplied the values by 255 for visualization of the spectrum. The normalization performed on the individual elements of the power spectrum is reported in Equation 1:

(1) $$V_{n}=255\times \left|V\right|\text{​}/V_{\text{max}}\times {\left(530\right)}^{2}$$

where “*V*” is the power of each frequency, “*V*_max_” is the peak of power across all frequencies, and “*V*_*n*_” is the normalized value. Following this normalization we split the spectrum in two halves by discarding the external part of the spectrum, reflecting especially low frequencies. Note that this procedure also discards high frequency patterns along a single dimension of the image, which could be due for example to sampling (i.e, pixel alternation). We then computed the average power across high frequencies. Note that in Equation 1 the variable *V*_max_ invariably reflected the power peak in very low frequency (i.e., close to 0). Therefore the procedure we used reflects the high-to-low frequencies ratio.

^2^We include rotten-food in the natural items for these analyses because, even though it is food, its status of being rotten makes it no longer a food choice. However, as other natural nonfood items rotten-food maintains texture, olfactory and palatable features.

## Appendix

**Table A1:** **Table of the validation data and information for each single image in the database**.

**Picture ID**	**Picture code**	**Name**	**Freq.**	**English translat.**	**Cal. per 100 g**	**Taxon.**	**P. calorie content**	**P. distance from eatability**	**P. level of transf.**	**Val.**	**Famil.**	**Typic.**	**Ambig. of the image**	**Arous.**	**Spatial freq.**	**Bright.**	**Size**
**NATURAL-FOOD**																	
pict_197	NF_037	Fragola	6	Strawberry	27	F	18.6 (15.5) [15.0] *n* = 19	14.6 (19.4) [9.0] *n* = 18	1.1 (2.5) [0.0] *n* = 18	77.1 (22.0) [76.0] *n* = 19	67.1 (27.6) [73.5] *n* = 20	88.2 (23.5) [100.0] *n* = 19	0.0 (0.0) [0.0] *n* = 20	72.7 (26.7) [75.0] *n* = 19	0.0078	204.05	0.34
pict_213	NF_053	Mandorle	21	Almonds	603	S	67.5 (26.6) [72.0] *n* = 19	13.4 (24.3) [0.5] *n* = 20	10.8 (20.6) [0.0] *n* = 18	70.5 (21.5) [66] *n* = 17	56.6 (31.2) [57.5] *n* = 18	82.7 (23.1) [87.5] *n* = 20	6.6 (22.7) [0.0] *n* = 19	31.0 (31.4) [18.0] *n* = 20	0.0033	188.83	0.51
pict_236	NF_076	Peperone	14	Pepper	26	V	22.1 (17.5) [23.0] *n* = 21	48.3 (26.6) [50.0] *n* = 21	3.3 (10.9) [0.0] *n* = 20	58.0 (27.1) [59.5] *n* = 18	64.0 (32.5) [66.0] *n* = 19	80.1 (23.2) [82.5] *n* = 18	0.2 (0.5) [0.0] *n* = 18	28.2 (27.2) [18.0] *n* = 19	0.0023	174.93	0.66
**TRANSFORMED-FOOD**																	
pict_4	TF_004	Baci di dama	2	Baci di dama	520	D	84.1 (11.1) [85.0] *n* = 19	14.7 (29.1) [0.0] *n* = 19	65.4 (17.6) [66.0] *n* = 18	77.4 (16.8) [78.0] *n* = 19	42.2 (26.3) [50.5] *n* = 18	74.3 (29.8) [85.0] *n* = 20	6.7 (19.5) [0.0] *n* = 19	68.1 (28.7) [73.5] *n* = 18	0.0021	210.60	0.37
pict_41	TF_041	Cotechino e lenticchie	10	Pork sausage with lentils	329	MS	75.4 (17.0) [78.5] *n* = 18	17.1 (31.4) [0.0] *n* = 20	62.9 (21.1) [65.0] *n* = 19	54.4 (31.5) [50.0] *n* = 19	34.0 (29.0) [35.0] *n* = 19	73.2 (17.2) [74.0] *n* = 18	6.3 (11.0) [0.0] *n* = 19	41.9 (30.4) [42.0] *n* = 17	0.0048	168.12	0.62
pict_94	TF_094	Patate fritte	6	French-fries	137	R	73.5 (25.4) [81.0] *n* = 19	12.9 (23.6) [0.0] *n* = 18	37.2 (23.5) [32.0] *n* = 19	51.1 (29.8) [50.0] *n* = 19	53.4 (30.5) [56.0] *n* = 19	69.8 (28.1) [78.0] *n* = 19	4.4 (11.0) [0.0] *n* = 18	41.2 (36.4) [27.5] *n* = 18	0.0016	237.74	0.31
**ROTTEN-FOOD**																	
pict_747	RF_005	Banane marce	12	Rotten bananas	65	F	23.0 (21.0) [20.5] *n* = 18	54.9 (44.4) [52.0] *n* = 21	4.4 (17.1) [0.0] *n* = 19	4.5 (14.4) [0.0] *n* = 18	8.2 (16.6) [0.0] *n* = 19	18.1 (27.4) [3.5] *n* = 18	10.8 (23.9) [0.0] *n* = 18	18.6 (28.7) [0.0] *n* = 19	0.0063	176.55	0.37
pict_755	RF_013	Formaggio marcio	57	Moldy parmesan	392	A	48.3 (35.1) [64.0] *n* = 19	55.7 (40.6) [54.0] *n* = 18	32.3 (30.9) [35.0] *n* = 21	8.0 (10.3) [1.5] *n* = 18	20.3 (26.5) [7.5] *n* = 18	19.1 (22.7) [7.0] *n* = 19	13.4 (25.8) [0.0] *n* = 18	38.3 (39.7) [21.0] *n* = 20	0.0021	212.32	0.47
pict_759	RF_017	Mais marcio	12	Rotten corn	353	V	31.7 (26.2) [28.0] *n* = 18	45.9 (42.7) [36.5] *n* = 18	7.8 (23.5) [0.0] *n* = 19	6.1 (8.4) [2.0] *n* = 18	11.0 (15.5) [0.0] *n* = 18	14.4 (21.0) [7.0] *n* = 19	5.3 (9.0) [2.0] *n* = 19	29.2 (38.0) [2.0] *n* = 18	0.0029	215.16	0.45
**ARTIFICIAL FOOD-RELATED OBJECTS**																	
pict_661	AFO100	Taglia pizza	0	Pizza cutter	–	–	–	–	–	57.6 (23.2) [51.5] *n* = 20	35.8 (32.6) [21.0] *n* = 21	70.4 (25.2) [75.0] *n* = 19	6.2 (14.8) [0.0] *n* = 18	26.8 (31.2) [14.0] *n* = 19	0.0059	234.73	0.29
pict_670	AFO109	Tazzina	7	Teacup	–	–	–	–	–	63.9 (20.1) [53.5] *n* = 18	58.1 (39.8) [72.5] *n* = 20	72.4 (25.6) [81.0] *n* = 19	7.3 (20.9) [0.0] *n* = 18	17.0 (21.0) [12.5] *n* = 20	0.0018	228.54	0.48
pict_671	AFO110	Padella	6	Pan	–	–	–	–	–	65.9 (23.9) [63.5] *n* = 18	87.3 (14.9) [93.5] *n* = 20	90.9 (11.2) [95.0] *n* = 19	1.1 (2.6) [0.0] *n* = 18	26.0 (29.4) [14.0] *n* = 19	0.002	196.54	0.44
**NATURAL NON-FOOD**																	
pict_693	NNF009	Conchiglia	10	Shell	–	–	–	–	–	65.2 (19.6) [63.0] *n* = 21	27.0 (25.2) [23.0] *n* = 18	59.4 (31.8) [74.0] *n* = 19	2.2 (4.8) [0.0] *n* = 18	29.9 (29.5) [17.5] *n* = 18	0.0024	0.0024	235.31
pict_718	NNF034	Lana	46	Wool	–	–	–	–	–	65.4 (17.6) [62.5] *n* = 18	31.0 (30.7) [17.0] *n* = 21	70.7 (29.1) [77.0] *n* = 19	3.4 (9.2) [0.0] *n* = 19	13.2 (15.7) [9.0] *n* = 19	0.0058	0.0058	136.05
pict_729	NNF045	Pigna	2	Pinecone	–	–	–	–	–	50.5 (20.5) [50.0] *n* = 19	54.3 (30.2) [52.0] *n* = 20	83.3 (23.2) [95.0] *n* = 19	6.9 (21.1) [0.0] *n* = 18	11.9 (16.5) [2.0] *n* = 18	0.0042	0.0042	160.59
pict_275	AO_014	Aspira polvere	2	Vacuum cleaner	–	–	–	–	–	56.1 (27.6) [50.0] *n* = 19	75.6 (30.0) [88.5] *n* = 18	83.3 (21.7) [94.0] *n* = 19	6.7 (22.5) [0.0] *n* = 21	16.2 (16.6) [13.0] *n* = 18	0.0082	215.04	0.25
pict_331	AO_070	Chitarra	39	Guitar	–	–	–	–	–	74.3 (20.9) [79.5] *n* = 18	45.2 (38.0) [43.0] *n* = 19	78.4 (32.5) [100.0] *n* = 20	6.7 (23.1) [0.0] *n* = 18	51.9 (31.7) [56.5] *n* = 18	0.0038	222.47	0.31
pict_390	AO_129	Guanti	25	Gloves	–	–	–	–	–	65.1 (27.2) [64.5] *n* = 18	54.7 (30.3) [52.0] *n* = 19	73.4 (21.0) [79.5] *n* = 18	4.6 (16.4) [0.0] *n* = 19	13.8 (26.3) [0.0] *n* = 17	0.0129	188.49	0.96
**ANIMALS**																	
pict_789	A__003	Aquila	14	Eagle	–	–	–	–	–	53.9 (36.4) [59.0] *n* = 21	3.4 (7.1) [0.0] *n* = 20	63.0 (32.5) [70.0] *n* = 19	0.3 (1.3) [0.0] *n* = 19	68.0 (32.0) [77.5] *n* = 18	0.0112	204.52	0.25
pict_791	A__005	Cammello	11	Camel	–	–	–	–	–	56.7 (22.6) [62.5] *n* = 18	0.5 (1.2) [0.0] *n* = 19	60.2 (35.3) [69.0] *n* = 19	7.4 (23.0) [0.0] *n* = 18	43.4 (37.4) [49.5] *n* = 18	0.0115	217.86	0.42
pict_803	A__017	Farfalla	24	Butterfly	–	–	–	–	–	61.5 (26.0) [59.0] *n* = 19	46.1 (32.1) [43.5] *n* = 20	65.8 (24.1) [60.5] *n* = 20	9.5 (18.3) [0.0] *n* = 18	54.2 (25.8) [60.0] *n* = 19	0.0096	194.89	0.44
**SCENES**																	
pict_843	S__002	Pista da sci	82	Ski slope	–	–	–	–	–	67.5 (24.2) [66.0] *n* = 19	30.4 (30.3) [16.5] *n* = 18	71.6 (31.3) [82.0] *n* = 20	7.1 (17.5) [0.0] *n* = 19	66.7 (30.0) [69.5] *n* = 18	0.0065	201.96	0.48
pict_850	S__009	Teatro	442	Theater	–	–	–	–	–	71.4 (25.8) [69.0] *n* = 19	34.5 (29.8) [22.0] *n* = 21	80.0 (22.9) [88.0] *n* = 19	7.7 (18.5) [0.0] *n* = 18	55.5 (37.5) [58.5] *n* = 18	0.0117	141.18	0.64
pict_868	S__027	Aereoporto	113	Airport	–	–	–	–	–	70.7 (24.1) [71.5] *n* = 18	43.4 (29.2) [39.5] *n* = 18	77.9 (27.1) [95.0] *n* = 18	9.4 (23.6) [0.0] *n* = 19	51.0 (36.1) [62.5] *n* = 18	0.0095	182.78	0.63

Here are provided three exemplars for each category. Figure 1 displays the actual picture of the examples reported here. FRIDa and the relative validation data for the full database are available upon request from http://foodcast.sissa.it/neuroscience/

Picture ID: Unique image identification code within FRIDa database.

Picture code: Image code composed of the image category acronym and image number within the image category: e.g., NF_037 stands for the 37th image (037) of Natural food image category (NF).

Name: Italian name of the depicted item.

Freq.: Frequency of the name in language.

English Translat.: English name of the depicted item.

Cal. per 100 g: Actual amount of calories per 100 grams of the depicted food item.

Taxon.: Taxonomy of the depicted food item (e.g., A, animal derivates; D, dessert; F, fruit; M, meat; S, seeds; V, vegetable).

P. calorie content: Estimated calorie content of 100 g of the depicted food item. Mean, standard deviation (between brackets), median [between squared brackets], and number of raters.

P. distance from eatability: Estimated amount of work required to bring the depicted food item to an eatable state from 0 (very little work necessary) to 100 (a lot of work necessary). Mean, standard deviation (between brackets), median [between squared brackets], and number of raters.

P. level of transf.: Perceived level of transformation of the depicted food item operationalized by how much work was required to prepare the food represented in the image implying how elaborated the food is from 0 (no work at all was required) to 100 (a lot of work was required). Mean, standard deviation (between brackets), median [between squared brackets], and number of raters.

Val.: Valence rating of the depicted item expressing the pleasantness of the image from 0 (very negative) to 100 (very positive). Mean, standard deviation (between brackets), median [between squared brackets], and number of raters.

Famil.: Familiarity rating of the depicted item referring to the frequency with which a person reports encountering the item in his/her daily life from 0 (never) to 100 (often). Mean, standard deviation (between brackets), median [between squared brackets], and number of raters.

Typic.: Typicality rating of the depicted item within its own category (e.g., how typical is what is represented in the image of its category, e.g., how typical of a lion is the lion represented in the image) from 0 (not at all) to 100 (very much). Mean, standard deviation (between brackets), median [between squared brackets], and number of raters.

Ambig. of the image: Subjective difficulty of correctly identifying the subject of the image from 0 (very easy) to 100 (very difficult). Mean, standard deviation (between brackets), median [between squared brackets], and number of raters.

Arous.: Arousal experienced in viewing the image from 0 (not at all arousing) to 100 (extremely arousing). Mean, standard deviation (between brackets), median [between squared brackets], and number of raters.

Spatial freq.: Spatial frequency (see footnote 1 for calculation procedure).

Bright.: Mean brightness of the grayscale version of the image.

Size: Ratio between non-white pixels and total image size (for all image 530 × 530).

**Table A2:** **Table of the validation data and information for each single image in the database on the rating split by gender**.

**Picture ID**	**English translat**	**Female raters**								**Male raters**							
		**P. calorie content**	**P. distance from eatability**	**P. level of transf.**	**Val.**	**Famil.**	**Typic.**	**Ambig. of the image.**	**Arous.**	**P. calorie content**	**P. distance from eatability**	**P. level of transf.**	**Val.**	**Famil.**	**Typic.**	**Ambig. of the image.**	**Arous.**
**NATURAL-FOOD**																	
pict_197	Strawberry	17.3 (17.7) [9.5] *n* = 10	13.7 (18.7) [9.0] *n* = 10	1.8 (2.9) [0.0] *n* = 11	70.9 (26.7) [74.0] *n* = 9	72.5 (27.0) [79.5] *n* = 8	89.1 (13.0) [95.0] *n* = 10	0.0 (0.0) [0.0] *n* = 14	84.8 (17.1) [91.0] *n* = 8	20.1 (12.6) [17.0] *n* = 9	15.6 (20.2) [5.5] *n* = 8	0.0 (0.0) [0.0] *n* = 7	82.7 (14.6) [86.5] *n* = 10	63.5 (27.4) [71.0] *n* = 12	87.2 (31.2) [100.0] *n* = 9	0.0 (0.0) [0.0] *n* = 6	63.9 (28.8) [70.0] *n* = 11
pict_213	Almonds	69.7 (21.6) [71.0] *n* = 10	11.1 (14.6) [3.0] *n* = 11	3.9 (7.4) [0.0] *n* = 10	72.7 (20.6) [68.0] *n* = 9	61.6 (33.8) [60.0] *n* = 8	81.7 (13.5) [82.0] *n* = 9	9.2 (28.7) [0.0] *n* = 11	19.9 (24.6) [7.0] *n* = 7	65.1 (31.1) [72.0] *n* = 9	16.1 (32.3) [0.0] *n* = 9	19.5 (27.4) [1.5] *n* = 8	68.1 (22.4) [66.0] *n* = 8	52.6 (28.3) [55.5] *n* = 10	83.4 (28.6) [96.0] *n* = 11	3.1 (8.3) [0.0] *n* = 8	37.0 (32.9) [21.0] *n* = 13
pict_236	Pepper	22.3 (19.5) [21.0] *n* = 15	50.2 (21.5) [51.0] *n* = 11	0.8 (2.0) [0.0] *n* = 8	44.0 (25.6) [47.0] *n* = 6	66.9 (28.5) [66.0] *n* = 9	82.1 (19.3) [85.0] *n* = 11	0.3 (0.6) [0.0] *n* = 12	23.6 (27.5) [9.0] *n* = 10	21.5 (11.1) [23.5] *n* = 6	46.2 (31.1) [41.5] *n* = 10	5.0 (13.7) [0.0] *n* = 12	65.0 (25.0) [64.5] *n* = 12	61.4 (35.6) [66.0] *n* = 10	76.9 (28.0) [79.0] *n* = 7	0.0 (0.0) [0.0] *n* = 6	33.2 (26.1) [28.0] *n* = 9
**TRANSFORMED-FOOD**																	
pict_4	Baci di dama	86.0 (10.6) [85.0] *n* = 11	11.5 (22.4) [0.0] *n* = 8	70.3 (18.7) [70.5] *n* = 10	79.1 (19.1) [81.0] *n* = 13	43.3 (27.0) [47.0] *n* = 13	82.4 (19.1) [89.5] *n* = 12	9.3 (24.8) [0.0] *n* = 10	69.3 (30.0) [78.0] *n* = 11	81.5 (11.4) [80.5] *n* = 8	17.1 (32.9) [0.0] *n* = 11	59.4 (14) [56.5] *n* = 8	73.7 (9.3) [72.0] *n* = 6	39.2 (24.1) [54.0] *n* = 5	62.0 (37.8) [78.0] *n* = 8	3.9 (10.3) [0.0] *n* = 9	66.1 (26.4) [69.0] *n* = 7
pict_41	Pork sausage with lentils	75.4 (16.5) [78.5] *n* = 10	24.3 (38.3) [0.0] *n* = 11	72.2 (19.8) [69.0] *n* = 10	52.8 (33.1) [45.5] *n* = 12	42.7 (27.8) [55.5] *n* = 12	73.6 (15.0) [69.5] *n* = 8	6.2 (9.7) [0.0] *n* = 10	30.0 (32.1) [18.5] *n* = 8	75.4 (17.7) [77.5] *n* = 8	8.2 (15.9) [0.0] *n* = 9	52.6 (17.3) [51.0] *n* = 9	57.1 (28.5) [72.0] *n* = 7	19.1 (24.7) [6.0] *n* = 7	72.9 (18.8) [74.5] *n* = 10	6.3 (12.3) [0.0] *n* = 9	52.4 (24.4) [50.0] *n* = 9
pict_94	French-fries	87.8 (12.1) [90.0] *n* = 6	25.6 (28.1) [19.0] *n* = 9	33.7 (22.0) [28.0] *n* = 13	53.4 (31.9) [55.0] *n* = 11	58.1 (27.6) [60.0] *n* = 14	65.1 (28.5) [72.0] *n* = 10	0.0 (0.0) [0.0] *n* = 9	50.6 (39.9) [66.0] *n* = 9	66.9 (27.2) [74.0] *n* = 13	0.2 (0.6) [0.0] *n* = 9	44.8 (24.8) [37.0] *n* = 6	48.0 (26.3) [45.5] *n* = 8	40.2 (34.4) [26.0] *n* = 5	75.1 (26.6) [79.0] *n* = 9	8.8 (14.3) [0.0] *n* = 9	31.9 (29.6) [17.0] *n* = 9
**ROTTEN-FOOD**																	
pict_747	Rotten bananas	23.7 (16.2) [22.0] *n* = 6	56.1 (42.8) [52.0] *n* = 11	7.9 (23.0) [0.0] *n* = 10	8.4 (19.5) [0.0] *n* = 9	3.7 (7.5) [0.0] *n* = 14	6.1 (13.6) [0.0] *n* = 12	3.8 (6.2) [0.0] *n* = 10	14.6 (25.9) [0.0] *n* = 12	22.7 (23.0) [14.5] *n* = 12	53.6 (46.0) [60.0] *n* = 10	0.4 (0.7) [0.0] *n* = 9	0.6 (1.6) [0.0] *n* = 9	20.6 (25.9) [0.0] *n* = 5	42.0 (32.1) [50.0] *n* = 6	19.6 (33.1) [0.0] *n* = 8	25.6 (31.8) [0.0] *n* = 7
pict_755	Moldy parmesan	53.0 (38.8) [69.0] *n* = 12	55.3 (35.5) [53.5] *n* = 8	42.1 (28.4) [50.0] *n* = 7	5.4 (6.9) [0.0] *n* = 9	32.9 (30.5) [23.0] *n* = 9	9.8 (11.4) [7.0] *n* = 11	10.6 (22.2) [0.0] *n* = 10	33.6 (34.2) [24.0] *n* = 9	40.1 (25.9) [30.0] *n* = 7	56.0 (44.2) [70.0] *n* = 10	27.4 (30.9) [20.5] *n* = 14	10.6 (12.3) [3.0] *n* = 9	7.7 (12.6) [0.0] *n* = 9	31.9 (27.7) [32.0] *n* = 8	17.0 (29.4) [3.0] *n* = 8	42.2 (43.3) [18.0] *n* = 11
pict_759	Mais marcio	36.1 (25.9) [38.5] *n* = 10	52.7 (41.8) [59.0] *n* = 11	4.4 (12.6) [0.0] *n* = 9	3.8 (7.1) [0.0] *n* = 10	12.8 (16.8) [1.0] *n* = 12	8.4 (14.2) [4.5] *n* = 14	3.1 (4.6) [0.0] *n* = 9	19.7 (32.1) [0.0] *n* = 12	26.1 (25.6) [17.5] *n* = 8	35.1 (42.0) [21.0] *n* = 7	10.8 (29.8) [0.0] *n* = 10	9.0 (9.1) [6.5] *n* = 8	7.3 (11.9) [0.0] *n* = 6	31.2 (27.0) [34.0] *n* = 5	7.3 (11.2) [3.0] *n* = 10	48.3 (41.4) [51.0] *n* = 6
**ARTIFICIAL FOOD-RELATED OBJECTS**																	
pict_661	Pizza cutter	–	–	–	59.0 (31.4) [48.0] *n* = 5	31.1 (25.7) [21.0] *n* = 13	82.7 (18.3) [89.0] *n* = 10	4.7 (8.5) [0.0] *n* = 11	15.5 (31.2) [2.5] *n* = 8	–	–	–	57.1 (19.7) [52.0] *n* = 15	43.4 (40.2) [34.5] *n* = 8	56.8 (24.7) [54.0] *n* = 9	8.6 (21.0) [0.0] *n* = 7	35.1 (28.4) [27.0] *n* = 11
pict_670	Teacup	–	–	–	68.2 (22.4) [51.0] *n* = 12	82.0 (27.8) [94.0] *n* = 12	72.2 (25.4) [81.0] *n* = 13	0.5 (1.4) [0.0] *n* = 11	22.4 (25.7) [14.0] *n* = 10	–	–	–	55.3 (10.1) [56.0] *n* = 6	22.3 (25.4) [15.5] *n* = 8	72.7 (25.9) [82.5] *n* = 6	18.0 (30.6) [0.0] *n* = 7	11.5 (12.8) [7.0] *n* = 10
pict_671	Pan	–	–	–	66.8 (26.3) [63.5] *n* = 12	86.1 (15.1) [91.0] *n* = 13	91.6 (11.9) [97.5] *n* = 8	1.2 (2.6) [0.0] *n* = 10	21.2 (29.9) [3.0] *n* = 13	–	–	–	64.2 (17.9) [58.0] *n* = 6	89.6 (14.1) [100.0] *n* = 7	90.4 (10.7) [95.0] *n* = 11	1.0 (2.7) [0.0] *n* = 8	36.3 (25.4) [33.0] *n* = 6
**NATURAL NON-FOOD**																	
pict_693	Shell	–	–	–	64.8 (17.0) [60.0] *n* = 12	25.2 (23.4) [23.0] *n* = 14	70.0 (22.9) [78.0] *n* = 9	1.5 (3.9) [0.0] *n* = 12	32.7 (29.7) [19.5] *n* = 12	–	–	–	65.8 (22.6) [66.0] *n* = 9	33.3 (29.7) [26.5] *n* = 4	49.8 (35.4) [50.0] *n* = 10	3.7 (5.9) [0.0] *n* = 6	24.5 (28.2) [10.0] *n* = 6
pict_718	Wool	–	–	–	71.8 (18.6) [70.0] *n* = 11	32.3 (32.0) [12.0] *n* = 13	71.2 (23.0) [76.0] *n* = 11	0.4 (1.0) [0.0] *n* = 8	11.1 (16.1) [0.0] *n* = 9	–	–	–	55.3 (8.9) [58.0] *n* = 7	28.9 (28.5) [22.5] *n* = 8	70.0 (35.8) [82.0] *n* = 8	5.6 (11.5) [0.0] *n* = 11	15.0 (15.1) [13.0] *n* = 10
pict_729	Pinecone	–	–	–	45.6 (20.6) [50] *n* = 12	50.6 (31.0) [42.5] *n* = 10	80.4 (20.6) [89.0] *n* = 9	2.8 (5.6) [0.0] *n* = 10	9.3 (15.2) [1.0] *n* = 12	–	–	–	58.9 (17.2) [52.0] *n* = 7	58.0 (28.9) [62.5] *n* = 10	85.8 (25.0) [100.0] *n* = 10	12.1 (30.2) [0.0] *n* = 8	17.2 (17.8) [14.5] *n* = 6
**ARTIFICIAL OBJECTS**																	
pict_275	Vacuum cleaner	–	–	–	61.5 (32.2) [57.0] *n* = 10	80.5 (23.4) [88.5] *n* = 12	89.1 (10.8) [92.0] *n* = 10	10.0 (30.0) [0.0] *n* = 10	16.9 (16.4) [13] *n* = 10	–	–	–	50.1 (19.6) [50.0] *n* = 9	65.7 (38.2) [81.0] *n* = 6	76.9 (28.0) [94.0] *n* = 9	3.6 (11.5) [0.0] *n* = 11	15.3 (16.9) [8.5] *n* = 8
pict_331	Guitar	–	–	–	68.3 (23.0) [71.0] *n* = 10	44.1 (37.3) [37.0] *n* = 9	81.5 (29.0) [100.0] *n* = 11	12.5 (33.1) [0.0] *n* = 8	50.8 (30.8) [55.0] *n* = 12	–	–	–	81.9 (14.7) [81.5] *n* = 8	46.2 (38.6) [46.5] *n* = 10	74.6 (35.9) [100.0] *n* = 9	2.0 (6.0) [0.0] *n* = 10	54.2 (33.2) [59.0] *n* = 6
pict_390	Gloves	–	–	–	70.4 (28.2) [68.0] *n* = 9	61.1 (33.5) [73.0] *n* = 10	82.4 (21.5) [91.0] *n* = 8	10.4 (25.5) [0.0] *n* = 7	20.2 (30.7) [3.0] *n* = 9	–	–	–	59.7 (25.1) [61.0] *n* = 9	47.6 (24.4) [41] *n* = 9	66.2 (17.6) [69.0] *n* = 10	1.2 (3.9) [0.0] *n* = 12	6.6 (17.5) [0.0] *n* = 8
**ANIMALS**																	
pict_789	Eagle	–	–	–	36.0 (30.6) [32.5] *n* = 10	0.7 (2.1) [0.0] *n* = 10	65.6 (30.4) [67.0] *n* = 9	0.6 (1.8) [0.0] *n* = 10	59.5 (35.2) [58.0] *n* = 11	–	–	–	70.2 (33.5) [91.0] *n* = 11	6.1 (9.0) [1.5] *n* = 10	60.7 (34.1) [72.0] *n* = 10	0.0 (0.0) [0.0] *n* = 9	81.4 (19.9) [92.0] *n* = 7
pict_791	Camel	–	–	–	54.0 (22.1) [63.0] *n* = 11	0.3 (0.9) [0.0] *n* = 9	63.1 (36.0) [80.0] *n* = 7	17.7 (36.9) [0.0] *n* = 6	46.0 (35.3) [56.0] *n* = 9	–	–	–	61.0 (22.6) [62.0] *n* = 7	0.7 (1.4) [0.0] *n* = 10	58.4 (34.8) [60] *n* = 12	2.3 (5.8) [0.0] *n* = 12	40.8 (39.1) [43.0] *n* = 9
pict_803	Butterfly	–	–	–	67.8 (20.4) [68.0] *n* = 8	39.6 (26.7) [39.0] *n* = 9	78.8 (19.8) [86.0] *n* = 9	12.4 (22.9) [0.0] *n* = 5	51.4 (28.1) [60.5] *n* = 8	–	–	–	57.0 (28.5) [58.0] *n* = 11	51.4 (35.0) [50.0] *n* = 11	55.1 (21.9) [56.0] *n* = 11	8.4 (16.0) [0.0] *n* = 13	56.3 (23.8) [60.0] *n* = 11
pict_843	Ski slope	–	–	–	68.5 (23.8) [65.5] *n* = 10	39.8 (31.7) [36.0] *n* = 8	64.6 (28.9) [73.0] *n* = 11	10.2 (24.4) [0.0] *n* = 9	67.8 (25.4) [63.5] *n* = 10	–	–	–	66.4 (24.6) [70.0] *n* = 9	22.9 (26.9) [10.0] *n* = 10	80.1 (32.1) [99.0] *n* = 9	4.3 (5.7) [0.0] *n* = 10	65.4 (34.9) [79.5] *n* = 8
pict_850	Theater	–	–	–	70.9 (20.7) [61.0] *n* = 11	36.7 (34.7) [19.0] *n* = 10	80.9 (14.2) [88.0] *n* = 10	13.3 (23.3) [2.0] *n* = 10	56.9 (36.2) [64.0] *n* = 9	–	–	–	72.0 (31.6) [77.5] *n* = 8	32.5 (24.4) [45.0] *n* = 11	78.9 (29.7) [88.0] *n* = 9	0.8 (1.6) [0.0] *n* = 8	54.1 (38.6) [53.0] *n* = 9
pict_868	Airport	–	–	–	75.0 (16.7) [76.0] *n* = 7	56.3 (29.4) [62.5] *n* = 8	64.0 (30.6) [57.5] *n* = 8	19.4 (33.6) [0.0] *n* = 8	51.1 (38.5) [62.0] *n* = 8	–	–	–	68.0 (27.4) [70.0] *n* = 11	33.2 (24.6) [35.5] *n* = 10	89.1 (17.1) [100.0] *n* = 10	2.1 (4.7) [0.0] *n* = 11	50.9 (34.1) [62.5] *n* = 10

Here are provided three exemplars for each category. Figure 1 displays the actual picture of the examples reported here. FRIDa and the relative validation data for the full database are available upon request from http://foodcast.sissa.it/neuroscience/

Picture ID: Unique image identification code within FRIDa database.

English Translat.: English name of the depicted item.

P. calorie content: Estimated calorie content of 100 g of the depicted food item. Mean, standard deviation (between brackets), median [between squared brackets], and number of raters.

P. distance from eatability: Estimated amount of work required to bring the depicted food item to an eatable state from 0 (very little work necessary) to 100 (a lot of work necessary). Mean, standard deviation (between brackets), median [between squared brackets], and number of raters.

P. level of transf.: Perceived level of transformation of the depicted food item operationalized by how much work was required to prepare the food represented in the image implying how elaborated the food is from 0 (no work at all was required) to 100 (a lot of work was required). Mean, standard deviation (between brackets), median [between squared brackets], and number of raters.

Val.: Valence rating of the depicted item expressing the pleasantness of the image from 0 (very negative) to 100 (very positive). Mean, standard deviation (between brackets), median [between squared brackets], and number of raters.

Famil.: Familiarity rating of the depicted item referring to the frequency with which a person reports encountering the item in his/her daily life from 0 (never) to 100 (often). Mean, standard deviation (between brackets), median [between squared brackets], and number of raters.

Typic.: Typicality rating of the depicted item within its own category (e.g., how typical is what is represented in the image of its category, e.g., how typical of a lion is the lion represented in the image) from 0 (not at all) to 100 (very much). Mean, standard deviation (between brackets), median [between squared brackets], and number of raters.

Ambig. of the image: Subjective difficulty of correctly identifying the subject of the image from 0 (very easy) to 100 (very difficult). Mean, standard deviation (between brackets), median [between squared brackets], and number of raters.

Arous.: Arousal experienced in viewing the image from 0 (not at all arousing) to 100 (extremely arousing). Mean, standard deviation (between brackets), median [between squared brackets], and number of raters.
